# Is a Water Content of 60% Maximum Water Holding Capacity Suitable for *Folsomia candida* Reproduction Tests? A Study with Silver Nanoparticles and AgNO_3_

**DOI:** 10.3390/ijerph15040652

**Published:** 2018-04-01

**Authors:** Moira S. McKee, Amelia Megía Guerrero, Juliane Filser

**Affiliations:** University of Bremen, UFT, FB02, General and Theoretical Ecology, Leobener Str. 6, 28359 Bremen, Germany; Amelia.MegiaGuerrero@haw-hamburg.de (A.M.G.); filser@uni-bremen.de (J.F.)

**Keywords:** soil moisture, OECD guideline 232, NM-300K

## Abstract

Silver nanoparticles (AgNP) are increasingly emitted to the environment due to a rise in application in various products; therefore, assessment of their potential risks for biota is important. In this study the effects of AgNP at environmentally relevant concentrations (0.6–375 µg kg^−1^ soil) on the soil invertebrate *Folsomia candida* in OECD (Organisation for Economic Co-operation and Development) soil was examined at different soil water contents. Animals were retrieved by heat extraction, which had an efficiency of about 90% compared with the floatation method. The tested water content range is set by OECD Guideline 232 (40–60% of the maximum water holding capacity, WHC), and we detected significant differences in toxicity due to these. With AgNO_3_, used as an ionic control, the number of juveniles significantly decreased only at 40% WHC, which might be due to dilution of the toxicant at higher soil water content. In turn, at 60% WHC, the reproduction of *F. candida* significantly increased in the presence of AgNP compared with in the control. However, at this water content, the required number of juveniles in the control treatment was not reached in three independent tests. The fact that the OECD validity criterion is not met indicates that the soil conditions are not suitable for reproduction at 60% WHC.

## 1. Introduction

In recent years the use of silver nanoparticles (AgNP) has become increasingly widespread across many areas of application. These range from cosmetics and medical technologies to construction materials and coatings of textiles [1,2]. The main characteristic for which AgNP are applied is their antimicrobial effects. AgNP can be emitted during production, use, and disposal of such products and therefore pose a risk to the environment. Modelling of AgNP life cycles predicts that major proportions of emissions will end up in soils. Currently the environmental concentrations are still very low, for example, 20–350 ng·kg^−1^ [3] in Denmark, but are expected to increase. Numerous studies have examined the toxicity of AgNP to soil invertebrates and detected negative effects on reproduction, survival, biomass, and gene expression (e.g., [4,5,6,7,8,9]). Species-, particle-, and concentration-dependent effects were found. It was also detected that soil properties influence the behavior and, consequently, the toxicity of AgNP in soil. The pH, organic matter content, ionic strength, and clay content have been identified as important factors in this context [9,10,11,12,13,14]. This means that depending on the characteristics of the soil used in a study, the observed effects of AgNP might vary, causing a different risk assessment of this new contaminant.

To standardize toxicity testing across institutes around the world, the OECD (Organisation for Economic Co-operation and Development) has established guidelines for toxicity testing of chemicals. It has been agreed upon by various experts that there is a need to amend the existing guidelines to make them more suitable for the testing of nanomaterials [15,16]. In a meeting for the OECD Working Party on Manufactured Nanomaterials, it was discussed that most OECD guidelines for ecotoxicological testing are generally applicable to the use of nanomaterials, yet the experts also identified a long list of open questions and research needs [15]. OECD Guideline 232 in this study specifies that the composition of an artificially mixed soil used for testing the collembolan *Folsomia candida* should be 74% sand, 20% kaolin clay, and 5% peat and the water content should be adjusted to 40 to 60% of the maximum water holding capacity of the soil [17].

The aim of this study is to test whether the soil water content influences the toxicity of the standard AgNP, NM-300K, and AgNO_3_ in OECD soil. We propose that the bioavailability of Ag changes when the water content in the soil changes, which can affect its toxicity to *F. candida*. Low concentrations in the µg range are tested to ensure environmental relevance.

## 2. Materials and Methods

### 2.1. Chemicals

AgNP NM-300K were obtained from the Joint Research Center as they are recommended for testing of nanomaterial hazards by the European Commission and the OECD [18]. NM-300K contains 10.16% *w*/*w* Ag in nano form and is stabilized by the agents polyoxyethylene glycerol trioleate and polyoxyethylene (20) sorbitan mono-laurat (Tween 20), each 4% *w*/*w*, which prevents particle aggregation because of sterical repulsion [19]. From this solution, a 2% *w*/*w* stock solution in deionized water was prepared. This vial (20 mL) was sonicated (Bandelin, Sonorex AK100H, Mörfelden-Walldorf, Germany) for 15 min before a 100 mg L^−1^ concentration was prepared for the test. All NM-300K solutions were stored in the dark at room temperature. Additionally, a NM-300K dispersion without silver was purchased from the Joint Research Center and stored in the same way as NM-300K.

For characterization, we summarize the findings of Klein et al. (2011) [18] and Köser et al. (2017) [7]; for further details, refer to these publications. NM-300K are about 15 nm in size and 99% of the silver particles are below 20 nm [18]. In demineralized water, NM-300K particles (10 mg Ag L^−1^) have an average hydrodynamic diameter of about 40 nm and a zeta potential of −15 mV [20]. The 10 mg Ag L^−1^ NM-300K dispersion was colloidally stable for 14 days [20].

AgNO_3_ powder (≥99.0% purity, Sigma-Aldrich, Munich, Germany) was used as an ionic control and the stock solution (100 mg Ag L^−1^) was prepared immediately before application to the soils with demineralized, filtered water.

### 2.2. Toxicity Test

The miniaturized form [21] of OECD Guideline 232 for testing chemical toxicity was applied. For this, four 9- to 12-day-old animals were introduced to 10 g (dry mass) previously spiked and thoroughly mixed OECD soil. The pH of the soil was adjusted to 6.1 with CaCO_3_. AgNP were tested at 0.6, 3, 15, 75, and 375 µg Ag·kg soil^−1^ AgNP, and AgNO_3_ was applied at 0.6, 15, and 375 µg Ag·kg soil^−1^. The dispersant controls contained the same amount of NM-300K dispersant as the 0.6, 15, and 375 µg kg^−1^ treatments. All these treatments were performed at 40, 50, and 60% maximum water holding capacity (WHC) each and were replicated 5 times. A water control of each soil water content was replicated 8 times. In addition to this test, the controls with three different water treatments were independently conducted at two other points in time. During the test, the temperature was set to 20 °C and the light/dark rhythm in the incubator was 12:12 h. Bakers’ yeast (Dr. Oetker, Bielefeld, Germany) was provided as food at the test start and added again after 14 days. The water content was checked gravimetrically in a weekly rhythm and adjusted when needed. At the end of the 28-day reproduction test, a dynamic heat extraction with a MacFadyen Extractor (ecoTech Umwelt-Meßgeräte GmbH, Bonn, Germany) was performed. The temperature regime was set to increase by 5 °C every 12 h, starting at 25 °C and ending at 40 °C. After the extraction into ethylene glycol, the animals were counted under a stereoscope (Olympus SZH10 Research Stero, Tokio, Japan).

### 2.3. Pre-Test of Extraction Method

In a pre-test, the extraction efficiency of the heat extraction was identified. The goal was to establish whether the water content influenced the extraction efficiency. Control treatments of 40, 50, and 60% WHC were prepared the same way as described above and after the 28 day test a dynamic heat extraction was performed. The abovementioned temperature regime was applied and an additional floatation extraction was done with the soil after the heat extraction.

### 2.4. Data Analysis

In each sample, the number of juveniles and adults was determined at the end of the test. Statistical analyses were performed with R Studio (version 1.1.383, RStudio Inc., Boston, MA, USA). The data was first checked for normality with the Shapiro-Wilk test (*p* > 0.05). If necessary and possible, the data was transformed and a one-way ANOVA and post hoc Dunnett test (*p* < 0.05) were applied. If this was not possible, Wilcoxon tests or Kruskal-Wallis tests with post hoc Dunn tests (*p* < 0.05) were used.

Additionally, interactions between the chemical applied (AgNP, AgNO_3_, dispersant, water control) and the soil water content were tested with a generalized linear model (*p* > 0.05). Here, a quasi-Poisson distribution was chosen.

## 3. Results

Adult mortality was not affected by any of the tested treatments; therefore, only data on *F. candida* reproduction is shown in the following.

**Figure 1:**
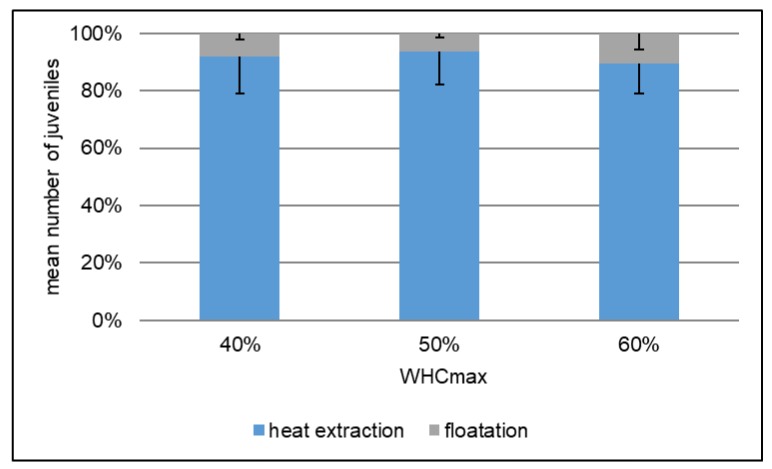
Extraction efficiency of the dynamic heat extraction at different soil water contents. Mean number of juveniles ± SE (*n* = 8) in untreated water controls after a 28 day reproduction test.

The extraction efficiency was not significantly different between treatments with the three different soil water contents. In all treatments the efficiency of the heat extraction was 90% of the total number of juveniles or higher (Figure 1).

**Figure 2:**
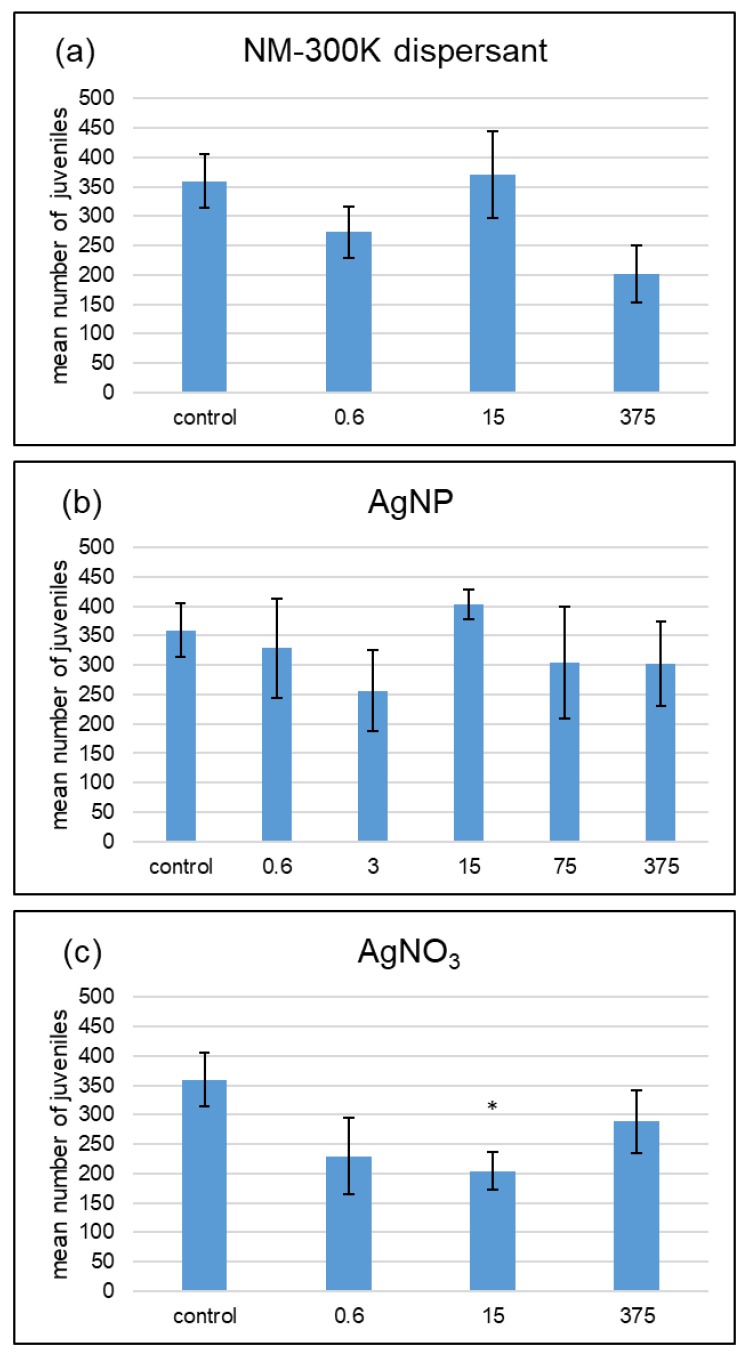
Effects of NM-300K dispersant (**a**), silver nanoparticles (AgNP) NM-300K (**b**), and AgNO_3_ (**c**) at 40% maximum water holding capacity on *F. candida* reproduction. Results of a 28-day reproduction test based on the miniaturized OECD (Organisation for Economic Co-operation and Development) test of Filser et al. (2014) [21]. The unit is µg Ag·kg^−1^ soil, and the dispersant treatments contain the same amount of dispersant as the AgNP treatment labeled with the same concentration. Asterisks indicate significant statistical differences to the respective control (Wilcoxon test, *p* > 0.05). Mean number of juveniles ± SE, *n* = 5; control: *n* = 8.

All but one treatment with 40% WHC did not show any significant difference in the number of juveniles to the control (Figure 2). Only soil with 15 µg Ag·kg^−1^ AgNO_3_ showed a significant decrease in juveniles compared with the control (Wilcoxon test). There was also significantly less reproduction in this AgNO_3_ treatment compared with the 15 µg Ag·kg^−1^ AgNP (Wilcoxon test).

**Figure 3:**
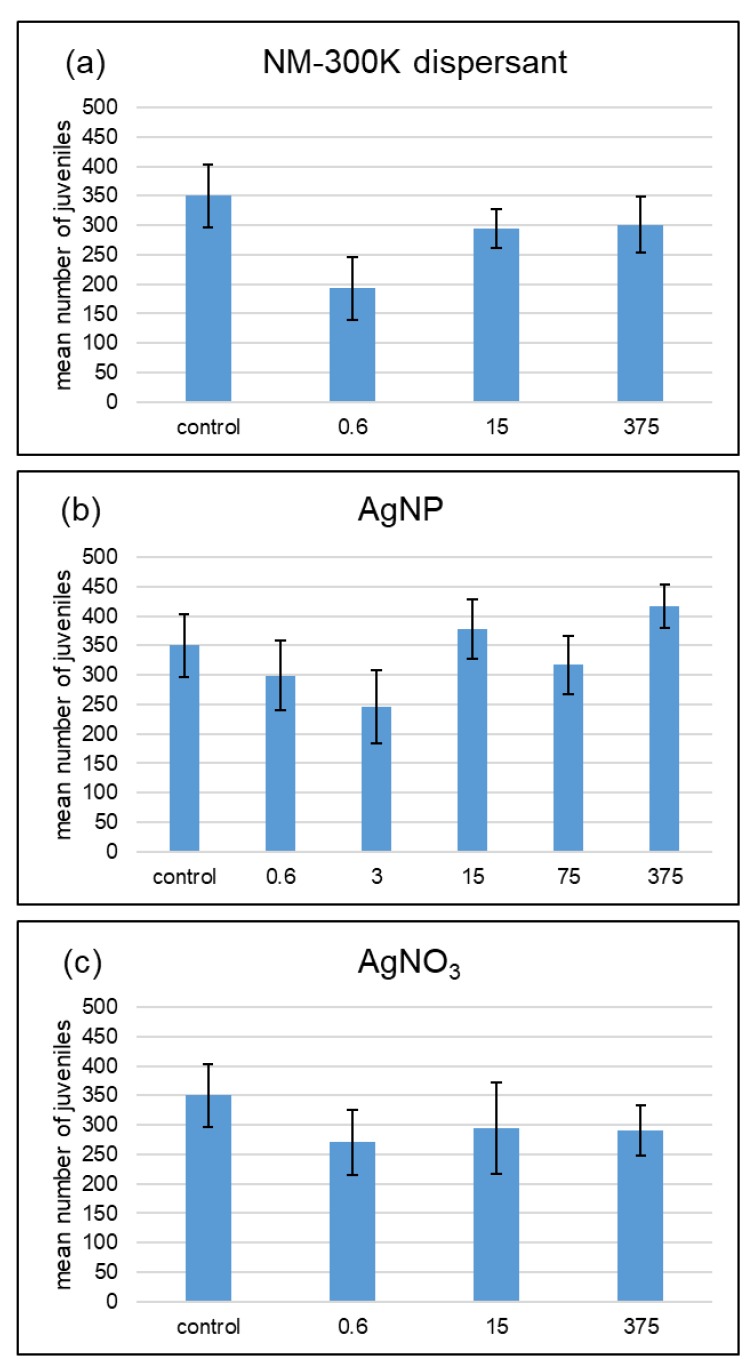
Effects of NM-300K dispersant (**a**), AgNP NM-300K (**b**), and AgNO_3_ (**c**) at 50% maximum water holding capacity on *F. candida* reproduction. Results of a 28-day reproduction test based on the miniaturized OECD test of Filser et al. (2014) [21]. The unit is µg Ag·kg^−1^ soil and the dispersant treatments contain the same amount of dispersant as the AgNP treatment labeled with the same concentration. Mean number of juveniles ± SE, *n* = 5; control: *n* = 8.

In the treatments with 50% WHC, none of the treatments with AgNP, dispersant, or AgNO_3_ were significantly different from the control (Figure 3).

**Figure 4:**
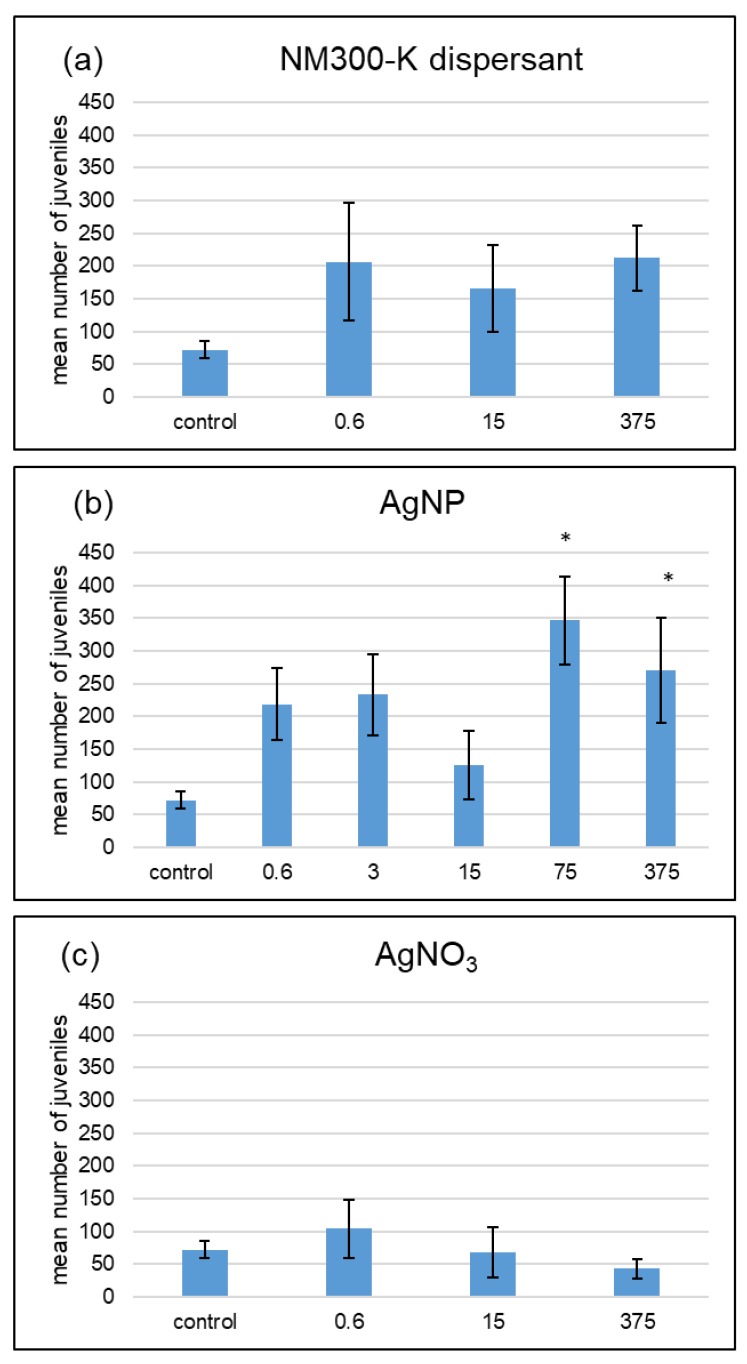
Effects of NM-300K dispersant (**a**), AgNP NM-300K (**b**), and AgNO_3_ (**c**) at 60% maximum water holding capacity on *F. candida* reproduction. Results of a 28-day reproduction test based on the miniaturized OECD test of Filser et al. (2014) [21]. The unit is µg Ag·kg^−1^ soil and the dispersant treatments have the same amount of dispersant as the AgNP treatment labeled with the same concentration. Asterisks indicate significant statistical differences to the respective control (Wilcoxon test, *p* > 0.05). Mean number of juveniles ± SE, *n* = 5; control: *n* = 8.

When the WHC was 60%, the two highest AgNP concentrations showed significant increases in reproduction compared with the control (Wilcoxon test). At the concentration of 375 µg Ag·kg^−1^, the treatment with AgNP had significantly more juveniles than the one with AgNO_3_ (Figure 4). A significant interaction between the chemical applied and the soil water content was detected (generalized linear model, *p* > 0.05).

**Figure 5:**
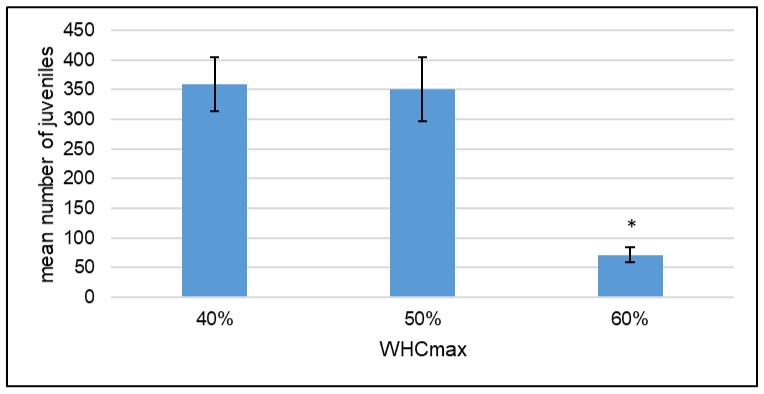
*F. candida* reproduction in the control treatments with different water contents. The mean number of juveniles after a 28 day reproduction test. The three treatments differ in percent of the maximum water holding capacity (WHCmax) of OECD soil. Mean ± SE, *n* = 8.

When comparing the controls of the three different water contents, a significant difference (Kruskal–Wallis and post hoc Dunn test) in number of juveniles between the 40%, 50%, and the much lower 60% WHC treatments becomes apparent (Figure 5). In contrast, the 40% and 50% are not significantly different from each other. This relationship was also detected in two other independent repetitions of the water controls with eight replicates each (data in Supplementary Figure S1). 

The other treatments were also compared between WHCs and no differences were found between 40% and 50% WHC in any treatment (Table 1). The number of juvenile *F. candida* was significantly lower in the 15 µg Ag·kg^−1^ AgNP treatment with 60% WHC than in the 40% and 50% WHC treatments (ANOVA and post hoc Dunnett test). The same was the case in the highest AgNO_3_ treatment. When 15 µg Ag·kg^−1^ AgNO_3_ was added, the treatment with 50% WHC had significantly higher reproduction than did the 60% treatment.

## 4. Discussion

The pre-test showed that the extraction efficiency of *F. candida* is independent of the water content of the OECD soil. Therefore, any observed differences between soil water contents can be attributed to the respective treatments and not to the methods applied.

### 4.1. No Dose-Response Curves for Silver Treatments

Within the tested range of concentrations of silver, no dose–response effect of silver was observed in any of the soil water treatments. This might be due to the low concentrations chosen for this toxicity assay which show low or no effects on the reproduction of *F. candida*. Other authors, who examined AgNP in a Collembola reproduction test, used much higher exposure concentrations and, depending on the particles and soil used, the EC_50_ values ranged from 540 to >1855 mg Ag·kg^−1^ [9,22,23]. The goal of the present study was, however, to focus on environmentally relevant concentrations to increase the realism of the conducted experiment [11]. This is not the first study to find no dose–response relationships when nanoparticles were applied. Simonin et al. (2017) [24] exposed ammonia-oxidizing bacteria and archaea as well as nitrite-oxidizing bacteria to low concentrations of TiO_2_NP and did not find an increase in toxicity with concentration for any of the microorganisms. In their study, several tests with low and high concentrations caused the highest toxicity. Studies with various cells, bacteria, and higher biological models have shown hormetic dose–responses to AgNP (reviewed by [25]). The increase of reproductive output in the 60% WHC treatment might also show hormesis from AgNP exposure. Further research is needed to assess the molecular mechanisms of hormesis caused by AgNP and other nanomaterials [25].

### 4.2. Different Effects of AgNP and AgNO_3_ with Different Soil Water Contents

Overall, the results of the reproduction test can be summarized as in Table 2. AgNP did not show any effects at 40% and 50% WHC, while they were significantly beneficial at 60% WHC with the tested concentrations. The dispersant did not significantly influence the reproduction of *F. candida* at any soil water content. AgNO_3_ significantly decreased reproduction at 40% WHC; however, this effect disappeared at higher water contents. The varying effects of the chemicals at different water contents were supported by a significant interaction between the tested chemical and soil moisture effect on *F. candida* reproduction.

At the two highest AgNP concentrations, the reproduction of *F. candida* increased at 60% WHC, which might be due to indirect effects within the soil community [11]. AgNP have antimicrobial effects and can therefore kill microorganisms in the soil that might be harmful to Collembola [26], freeing *F. candida* from pathogens and allowing higher reproduction. A second possibility is that the food availability for Collembola changed due to AgNP exposure in the soil [27]. Fungi are an important part of the collembolan diet and *F. candida* has been shown to be fungivorous [28]. Findings by Carbone et al. (2014) [29] indicate that bacteria are more sensitive to AgNP than fungi and it is known that fungi are more resistant to metal stress than bacteria [30]. This might cause higher reduction of bacteria than of fungi in AgNP-treated soil, possibly reducing the competition for fungi and releasing nutrients from dead bacteria for fungi to grow on. With an increase in fungi growth, Collembola would have more available food, allowing stronger reproduction. As the beneficial effect of AgNP to *F. candida* was only detected with the highest soil water content, this indicates that the potential indirect effects are stronger when more water is available. Bacteria proportions in soil microbial communities increase with higher water content while fungi thrive in soil with less water [31], which would cause a shift in the fungi/bacteria ratio, opposite of the described changes that would cause an increase in fungal food. However, due to the lack of information on the microbial community compositions in the applied test system, no clear explanation can be given for the observed effects. Further studies investigating these aspects are therefore needed. Overall, the favorable effects of AgNP in comparison with the control with 60% WHC in this test should not be overinterpreted because the test does not meet the OECD validity criteria. However, independent of whether these results can be used for official risk assessment, they give interesting new insights on the complexity of AgNP effects in soil systems.

The effect of AgNP at 375 mg Ag·kg^−1^ on reproduction is significantly different from the effect of AgNO_3_ at the same concentration in the 60% WHC treatment. A possible explanation for the lack of promotion of reproduction with AgNO_3_ might be that it is more toxic to *F. candida* than AgNP [9,22,23]. Additionally, the indirect effects described above might not be caused by AgNO_3_ because the ion dissolution from AgNP is slower and therefore has long-term antimicrobial effects while AgNO_3_ dissolves much faster [6]. Further chemical analyses of silver in OECD soil would be needed to support this possible explanation of the results.

AgNO_3_ was toxic at 40% WHC within the tested concentrations, while not at higher soil water contents. Dilution is higher in soil with a higher soil water content, which might cause decreased toxic effects. When van Gestel and van Diepen (1997) tested whether the soil water content affects cadmium toxicity to *F. candida*, they detected a difference in reproduction between 28 and 63% WHC. After six weeks, the number of juveniles increased with a decrease in soil water content. This difference was particularly pronounced at low concentrations [32], similar to the concentration range tested in the present study. Another possibility is that silver is spread around more in the soil when there is more pore water, which increases the likelihood of silver getting in contact with organic material to which it can bind [20]. Silver bound to organic material or other compounds in the soil is not as easily taken up by the collembolans and can therefore cause less toxicity [13]. Although at first sight contradictory, dilution also renders a hypothetical explanation as to why the AgNP effect was only observed at 60% WHC: Nanoparticles tend to agglomerate with increasing concentration, reducing their tendency to dissolve. Thus, at 60% WHC, more toxic action (in this case towards bacteria, see above) can be expected. Independently of what causes the dissimilarity in AgNP and AgNO_3_ effects, it is important to note that if the test would have been only performed with one of the three water contents, the assessment of toxicity would have been different even though the test was conducted in accordance with the OECD guideline.

### 4.3. Less Suitable Conditions for F. candida with 60% WHC

When comparing the reproduction independently of any contamination, it became apparent that *F. candida* reproduction was significantly higher in OECD soil when the soil water content was lower. This was detected in three independent repetitions of the control treatments with 40, 50, and 60% WHC. The mean number of juveniles was lower than 100 in the 60% WHC controls and therefore did not meet the validity criterion of the OECD guideline (OECD Guideline 232). *F. candida* is resistant to desiccation and adapted to dry soil conditions [28]. Soil aggregates are larger with a higher water content, which might cause anoxic conditions within these aggregates. Such conditions can be endured by *F. candida* for up to 18 h [28]; however, they might not allow reproduction. Also, the pore space available for Collembola to inhabit decreases with an increase in soil water content. Therefore density-dependent competition might become an issue, causing lower reproduction. It has been observed on charcoal culturing plates that when the water saturation is too high, Collembola hatching is less successful due to fungal and bacterial growth on the eggs (unpublished data). This might also take place in soil, further reducing the number of juveniles in OECD soil with a higher water content. Overall conditions seem less suitable for *F. candida* reproduction in soil with a higher water content and this might affect their stress tolerance also when testing other chemicals.

## 5. Conclusions

The soil water content influences the effects of AgNP NM-300K and AgNO_3_ in OECD soil in the range of tested, environmentally relevant concentrations. Investigating the reasons behind these differences should be the focus of further studies to assess how ecological and chemical parameters affect the bioavailability and toxicity of AgNP in complex soil matrices. The results of this study call attention to possible variability of results and, therefore, differences in risk assessment that can arise when soil is adjusted to different water contents.

## Figures and Tables

**Table 1:** Comparison of *F. candida* reproduction between treatments with different water content (percent of maximum water holding capacity). The unit is µg Ag·kg^−1^ soil and the dispersant treatments contain the same amount of dispersant as the AgNP treatment labeled with the same concentration. n.s. indicates no significant difference in the number of juveniles after the 28-day test. If there is a significant difference between the respective treatments, the *p*-value of the post hoc Dunnett test (*p >* 0.05) is shown. *n* = 5.

Treatment	Concentration (µg·kg^−1^)	40%–50%	40%–60%	50%–60%
AgNP	375	n.s.	n.s.	n.s.
AgNP	75	n.s.	n.s.	n.s.
AgNP	15	n.s.	0.003	0.007
AgNP	3	n.s.	n.s.	n.s.
AgNP	0.6	n.s.	n.s.	n.s.
dispersant	375	n.s.	n.s.	n.s.
dispersant	15	n.s.	n.s.	n.s.
dispersant	0.6	n.s.	n.s.	n.s.
AgNO_3_	375	n.s.	0.004	0.004
AgNO_3_	15	n.s.	n.s.	0.036
AgNO_3_	0.6	n.s.	n.s.	n.s.

**Table 2:** Summary of the effects of AgNP, AgNO_3_, and dispersant on *F. candida* reproduction with different soil water contents in the tested concentration range. ++ = strong increase, 0 = no difference, − = decrease.

Treatment	40% WHC	50% WHC	60% WHC
AgNP	0	0	++
AgNO_3_	−	0	0
dispersant	0	0	0

## Supplementary Materials

The following are available online at http://www.mdpi.com/1660-4601/15/4/652/s1, Figure S1: Reproduction in the controls with 40%, 50%, and 60% maximum water holding capacity (WHC).
