# Supplementary material for: Is a Water Content of 60% Maximum Water Holding Capacity Suitable for Folsomia candida Reproduction Tests? A Study with Silver Nanoparticles and AgNO3
Source: Int J Environ Res Public Health. 2018 Apr 1;15(4):652. doi: 10.3390/ijerph15040652 (PMC5923694; doi:10.3390/ijerph15040652)
Supplement: Supplementary file 1 [file ijerph-15-00652-s001.pdf]

Supplementary Materials

Reproduction in the controls

a)

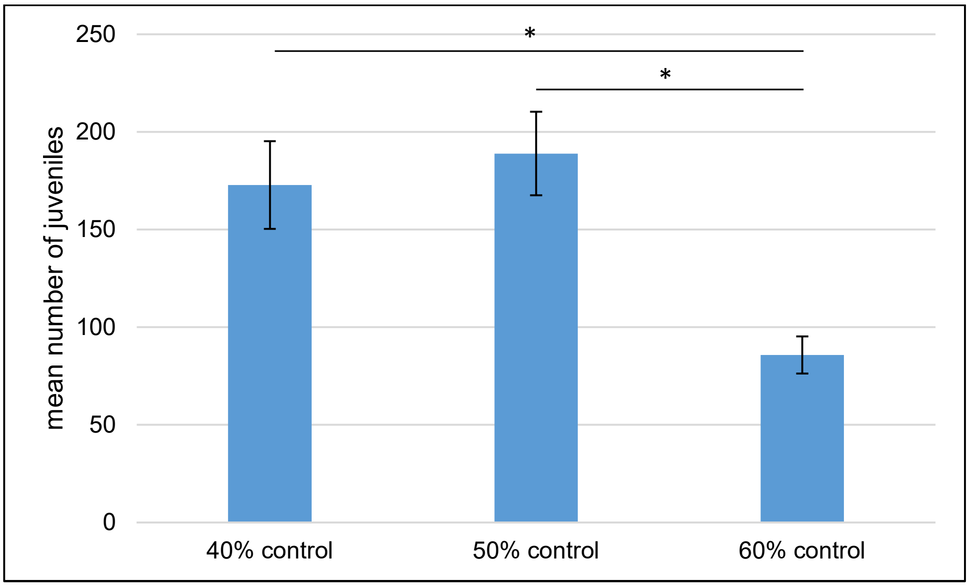

b)

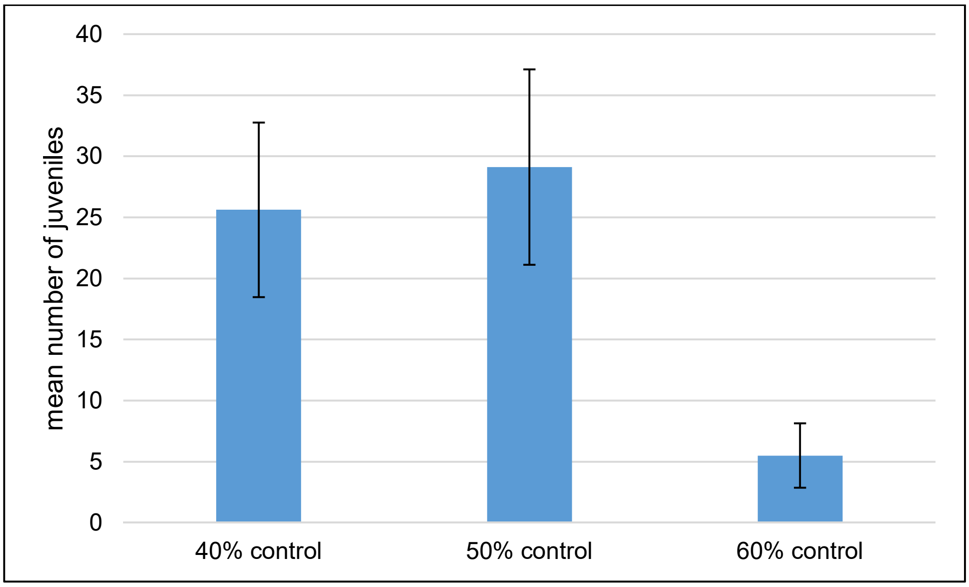

Figure S1: Reproduction in the controls with 40%, 50% and 60% maximum water holding capacity (WHC). *F. candida* reproduction in the control treatments with different water contents in two independent repetitions (a, b) in OECD soil (see Figure 4 for third repeat). Shown is the mean number of juveniles after a 28-day reproduction test. Asterisks indicate significant statistical differences between the respective controls (Kruskal-Wallis, post-hoc Dunn test, $p > 0.05$). Mean ± SE, n= 8.
